# MultiAlign: a multiple LC-MS analysis tool for targeted omics analysis

**DOI:** 10.1186/1471-2105-14-49

**Published:** 2013-02-12

**Authors:** Brian L LaMarche, Kevin L Crowell, Navdeep Jaitly, Vladislav A Petyuk, Anuj R Shah, Ashoka D Polpitiya, John D Sandoval, Gary R Kiebel, Matthew E Monroe, Stephen J Callister, Thomas O Metz, Gordon A Anderson, Richard D Smith

**Affiliations:** 1 Pacific Northwest National Laboratory, 99352, Richland, WA, USA

**Keywords:** Metabolomics, Proteomics, Mass spectrometry, Liquid chromatography, Spectral clustering, Alignment

## Abstract

**Background:**

MultiAlign is a free software tool that aligns multiple liquid chromatography-mass spectrometry datasets to one another by clustering mass and chromatographic elution features across datasets. Applicable to both label-free proteomics and metabolomics comparative analyses, the software can be operated in several modes. For example, clustered features can be matched to a reference database to identify analytes, used to generate abundance profiles, linked to tandem mass spectra based on parent precursor masses, and culled for targeted liquid chromatography-tandem mass spectrometric analysis. MultiAlign is also capable of tandem mass spectral clustering to describe proteome structure and find similarity in subsequent sample runs.

**Results:**

MultiAlign was applied to two large proteomics datasets obtained from liquid chromatography-mass spectrometry analyses of environmental samples. Peptides in the datasets for a microbial community that had a known metagenome were identified by matching mass and elution time features to those in an established reference peptide database. Results compared favorably with those obtained using existing tools such as VIPER, but with the added benefit of being able to trace clusters of peptides across conditions to existing tandem mass spectra. MultiAlign was further applied to detect clusters across experimental samples derived from a reactor biomass community for which no metagenome was available. Several clusters were culled for further analysis to explore changes in the community structure. Lastly, MultiAlign was applied to liquid chromatography-mass spectrometry-based datasets obtained from a previously published study of wild type and mitochondrial fatty acid oxidation enzyme knockdown mutants of human hepatocarcinoma to demonstrate its utility for analyzing metabolomics datasets.

**Conclusion:**

MultiAlign is an efficient software package for finding similar analytes across multiple liquid chromatography-mass spectrometry feature maps, as demonstrated here for both proteomics and metabolomics experiments. The software is particularly useful for proteomic studies where little or no genomic context is known, such as with environmental proteomics.

## Background

Liquid chromatography-tandem mass spectrometry (LC-MS/MS) analyses typically leverage database search tools [1-3] to accurately identify analytes (peptides, metabolites, etc.) from tandem mass spectra. In proteomics, search tools utilize a protein sequence database to generate *in silico* spectra that are statistically matched to the empirical spectra in order to assign the best amino acid sequence to the spectrum. These peptide sequences along with their mass and chromatographic elution time information are often stored in a reference peptide database that serves as a look up table for identifying peptides in subsequent higher-throughput, high-resolution LC-MS analyses [4]. The resulting LC-MS spectra are deisotoped using DeconTools [5] or similar software that applies algorithms such as THRASH [6]or RAPID [7] to obtain monoisotopic mass and elution time features. Once aligned, these features are matched to appropriate reference databases using software such as VIPER [8], MaxQuant [9], msInspect [10], mzMine [11], or SpecArray [12] for peptide identification and/or quantitation.

When a database is not available or is incomplete, as is often the case in environmental proteomics studies, peptide sequences are identified using alternative approaches, such as *de novo* sequencing or by combining multiple genomes to form a pseudo metagenome [13-16]. Reference peptide databases generated from these approaches tend to be error prone and poorly represent the sample proteome, which results in a substantial number of unidentified (unattributed) features and a failure to adequately capture proteome dynamics. Liquid chromatography-mass spectrometry-based global metabolomics studies are similarly challenged in that constructing a representative database for identifying metabolite features is difficult due to the lack of available metabolite standards, which often results in an incomplete identification.

Herein we introduce MultiAlign, a public domain software tool designed to overcome LC-MS data analysis challenges stemming from the lack of a reference database. With MultiAlign, multiple datasets are aligned by clustering mass and LC elution time features. In addition to revealing the presence/absence of features and differences in relative abundance from proteome or metabolome profiles, the software can cull a set of features for targeted peptide sequence or metabolite assignment via a traceback capability that links clusters of LC-MS features across numerous experimental runs to tandem mass spectra. This multi-alignment strategy is advantageous in that patterns among LC-MS global proteome profiles can be evaluated in the absence of reference databases, which is appealing to environmental proteomics applications, such as characterization of microbial communities that lack genome sequence information, as well as to other omics-based applications, such as metabolomics. While the comparative multi-dataset analysis approach is similar to that of XCMS [17], which is primarily used to process metabolomics data, MultiAlign is applicable to both proteomics and metabolomics data and allows for peak matching LC-MS data to reference databases (similar to VIPER [8]). Furthermore, MultiAlign supports full traceback from feature clusters to parent and corresponding tandem mass spectra.

## Implementation

MultiAlign is a multiple dataset analysis tool for clustering aligned LC-MS features (dataset to dataset or dataset to reference database, if available) across LC-MS experimental runs. Provided a reference database exists, these clusters can be matched to the database for peptide sequence assignment. MultiAlign also performs MS/MS spectral clustering to facilitate proteome structure characterization (i.e., types of peptides present in a system of biological samples).

### Architecture

**Figure 1:**
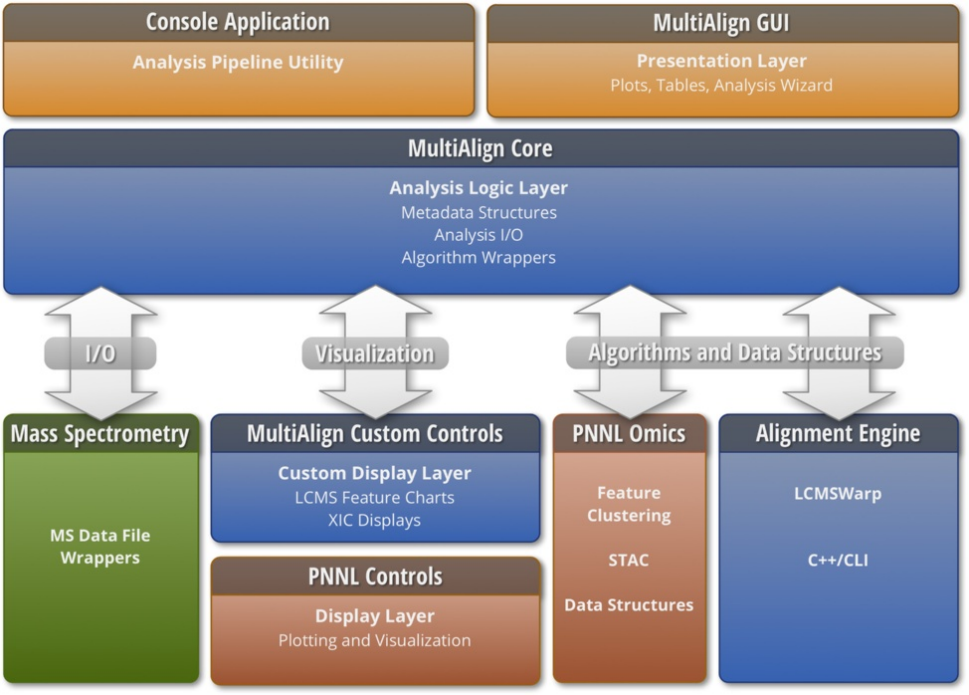
Architecture of the MultiAlign software.

The basic architecture of MultiAlign is depicted in Figure 1. The software is written on .NET framework version 4.0, using C# for the presentation layer, and a mixture of standard C++ (using the Standard Template Libraries) and C# for the computational engine. A C++/CLI adapter marshals data between C# and C++/CLI managed and unmanaged interfaces when features in the C++ engine are required. Results are persisted to a SQLite database. A graphical user interface is provided with the Microsoft Windows application version. Interactive feature scatter plots, heat maps, histograms, and line charts are created using a custom visualization package that can handle millions of data points by leveraging fast bitmapping techniques via GDI + library calls. A command line version is also supplied as an alternative to the user interface.

### Data structures

MultiAlign uses four simple data objects: MS features, LC-MS features, LC-MS feature clusters, and LC-MS/MS-generated accurate mass and normalized LC elution time features referred to as AMT tags. An MS feature represents the peaks of an isotopic distribution of peptide ions resulting from the deisotoping of an LC-MS spectrum using the THRASH algorithm [6]. Each MS feature is described by a monoisotopic mass, charge, and retention time. An LC-MS feature is a group of MS features common to a monoisotopic mass observed across elution time (i.e., in multiple consecutive spectra or scans), whereas an LC-MS feature cluster is composed of LC-MS features observed across multiple aligned datasets. An AMT tag feature is represented at the most basic level by monoisotopic mass, globally averaged normalized elution time (NET) between 0 and 1, and a peptide sequence. These features are stored in a reference peptide database referred to as an AMT tag database. Fragmentation spectra are represented by the MS^n^ spectra object by a precursor *m/z* linked to MS features within a given elution time.

### Algorithm

**Figure 2:**
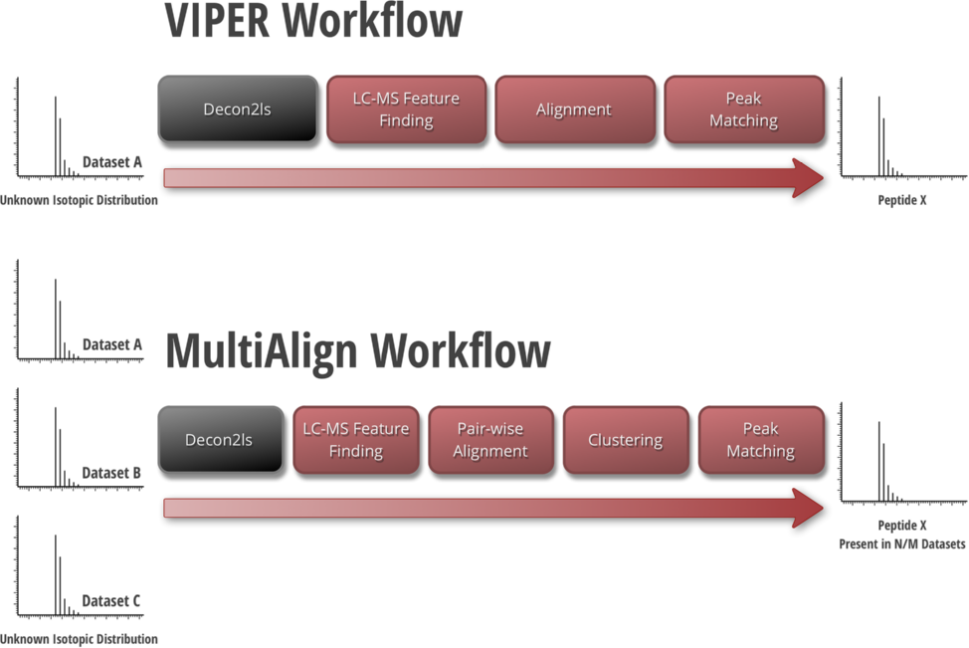
**The data workflow for MultiAlign is similar to that of its predecessor VIPER with the addition of the clustering step and the ability to perform multiple dataset analysis.** DeconTools is shown as the pre-processing step that reduces raw mass spectra into MS features of resolved monoisotopic masses, charge, and elution time.

Four major processing components exist in the computational layer: LC-MS feature finding, LC-MS feature alignment, LC-MS feature clustering, and peak matching to an AMT tag database. This computational workflow is similar to that of VIPER [8] (Figure 2), an existing LC-MS proteomics analysis tool, but with the addition of a clustering step that allows MultiAlign to perform dataset-to-dataset analysis in addition to finding unattributed features common across datasets. MultiAlign also provides a traceback capability that links LC-MS feature clusters to MS/MS spectra for use in peptide sequence assignment approaches that do not rely on database searches, e.g. *de novo* approaches.

### LC-MS feature finding

Peptides separated via LC are detected across several mass spectra at various charge states (when using electrospray ionization). Respective isotopic distributions of ionized peptides can be resolved to a single monoisotopic mass after normalizing by charge [4,5,8], which results in an MS feature. The feature finding component clusters these MS features into an LC-MS feature, using a mass tolerance window in parts per million (ppm) and assigns a representative LC scan based on the maximum signal intensity of the eluting LC peak. This clustering step uses a single linkage clustering algorithm with a weighted L^2^ norm distance calculation based on mass, elution time, and intensity; weights are user definable. Feature abundances are reported as the sum of monoisotopic peak values from all clustered MS features. The output database links feature data to scans from the instrument files, which allows researchers to develop custom quantitation algorithms to suite their specific applications.

When running on a hybrid instrument, MultiAlign will ingest raw data files (Thermo Finnigan binary or mzXML format) to link MS features found by THRASH to MS/MS spectra by searching daughter scans (MS level > 1) that have similar parent masses. The algorithm hashes all MS scans and then stores the MS/MS scan indices after which the precursor *m/z* from the MS/MS spectra is compared to the *m/z* of the MS feature. All matches are linked and stored in the results database. The end result is the ability to query all clusters and/or LC-MS features for which there is MS/MS spectra that can be exported to peptide identification tools, e.g., peptide spectrum match search and *de novo* approaches.

### Alignment

To correct for systematic variation in elution time and mass measurement accuracy, MultiAlign uses the LCMSWarp algorithm [18], a dynamic time warping approach. This algorithm computes a piecewise linear alignment function to calibrate the monoisotopic mass and transform scan number of each MS feature to the NET so that LC-MS features can be compared across datasets.

MultiAlign uses one of two alignment strategies. The first strategy is similar to VIPER [8] in that it aligns a single dataset to an AMT tag database. The second strategy, which is useful when no AMT tag database is available, aligns a dataset to another reference dataset. The analysis tool is capable of loading several datasets at once for subsequent pair-wise alignment.

MultiAlign also produces heat maps for visualizing how well a dataset aligns to a database or to a dataset. These heat maps display alignment probability scores between scans of the aligned and baseline data [18]. Ideally, one sees a bright linear yellow trend line from the lower left coordinate to the upper right, which indicates little retention time and mass variation between data.

### Clustering

If a peptide is represented across multiple LC-MS acquisitions obtained under similar conditions, then it should be visible as an LC-MS feature across all datasets. MultiAlign finds these common LC-MS features via average or single linkage clustering (specified by the user). Both algorithms initially treat each LC-MS feature across all datasets as a singleton cluster. These clusters are then iteratively merged based on minimum values in a distance map; single linkage merges are based on the shortest distance and average linkage merges, on average centroid distances. In the single linkage algorithm, clusters are merged only if they are within monoisotopic mass tolerance (ppm) and NET. These tolerances can be defined by the user based on knowledge of instrument performance. Clusters are assigned a score as to the mean L^2^ distance of each feature to the cluster centroid. This cluster score describes the overlap of LC-MS features in mass and retention time space across runs.

Both clustering algorithms use a simple mass partitioning approach to improve run time performance. The entire feature space from all datasets is sorted based on monoisotopic mass. Gaps in the list are identified based on the mass tolerance (ppm) between two consecutive features, which provides a 33-fold increase in speed and improves multiple dataset analysis runtime. Each cluster is assigned two scores, the first of which calculates the mean L^2^ distance of all features to the cluster centroid based on monoisotopic mass and NET. This score reflects tightness where the smaller the tightness, the more confident the cluster. The second score addresses ambiguity; that is, given any two clusters, what is the potential that they are indistinguishable by their dimensionality (e.g. monoisotopic mass or NET). If two clusters are close in monoisotopic mass and NET space, then the cluster is considered to be ambiguous. An ambiguity score is calculated as the minimum L^2^ norm distance between any two features from neighboring clusters. As the score approaches zero, the distance between pair wise members is small and indicates an ambiguous cluster. These scores serve as filtering metrics to find clusters of similarly eluting features across datasets in downstream analysis, e.g. presence/absence and relative quantitation.

### Peak matching

When an AMT tag database is available, MultiAlign performs peak matching to assign peptide sequences to LC-MS features. Peak matching is accomplished by using simple range queries with monoisotopic mass ppm and NET. However, a single LC-MS feature can be matched to multiple peptides, thus making the identification ambiguous. We utilize the STAC algorithm [19] to quantify the goodness of match for a cluster and an AMT tag in what is called the STAC score. The STAC algorithm also provides an orthogonal score (the STAC-UP score) that assesses the uniqueness probability of each match in the case of multiple AMT tags matching the same cluster, i.e., for a given cluster that matches to multiple mass tags, the uniqueness probability describes which match is the most likely.

### Spectral clustering

When MS/MS spectra are available, MultiAlign can cluster the spectra across multiple samples by implementing an algorithm similar to that of MSCluster [20] except that it incorporates retention time information at the MS feature level, which allows spectral databases to be constructed. This clustering method is a hierarchical approach analogous to that used for clustering LC-MS data. First, MS/MS spectra that belong to LC-MS features are considered for clustering, which limits the number of MS/MS spectra to be clustered. As spectra must have a representative elution profile (intensity vs. time) gained from the LC-MS feature finding step, the chance of transient MS/MS spectra (e.g., present in a single scan) entering the analysis is reduced. The MS/MS spectra are then compared across datasets, and the spectral similarity is computed as the normalized dot product of the ion series.

### Visualization

**Figure 3:**
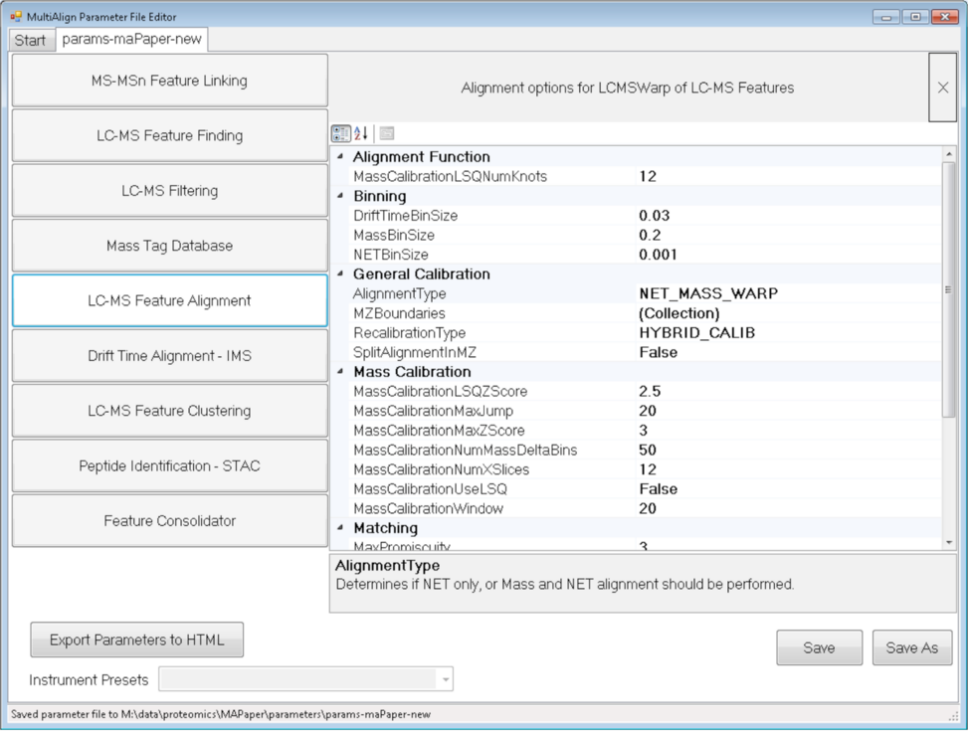
Standalone parameter file editor application screenshot.

**Figure 4:**
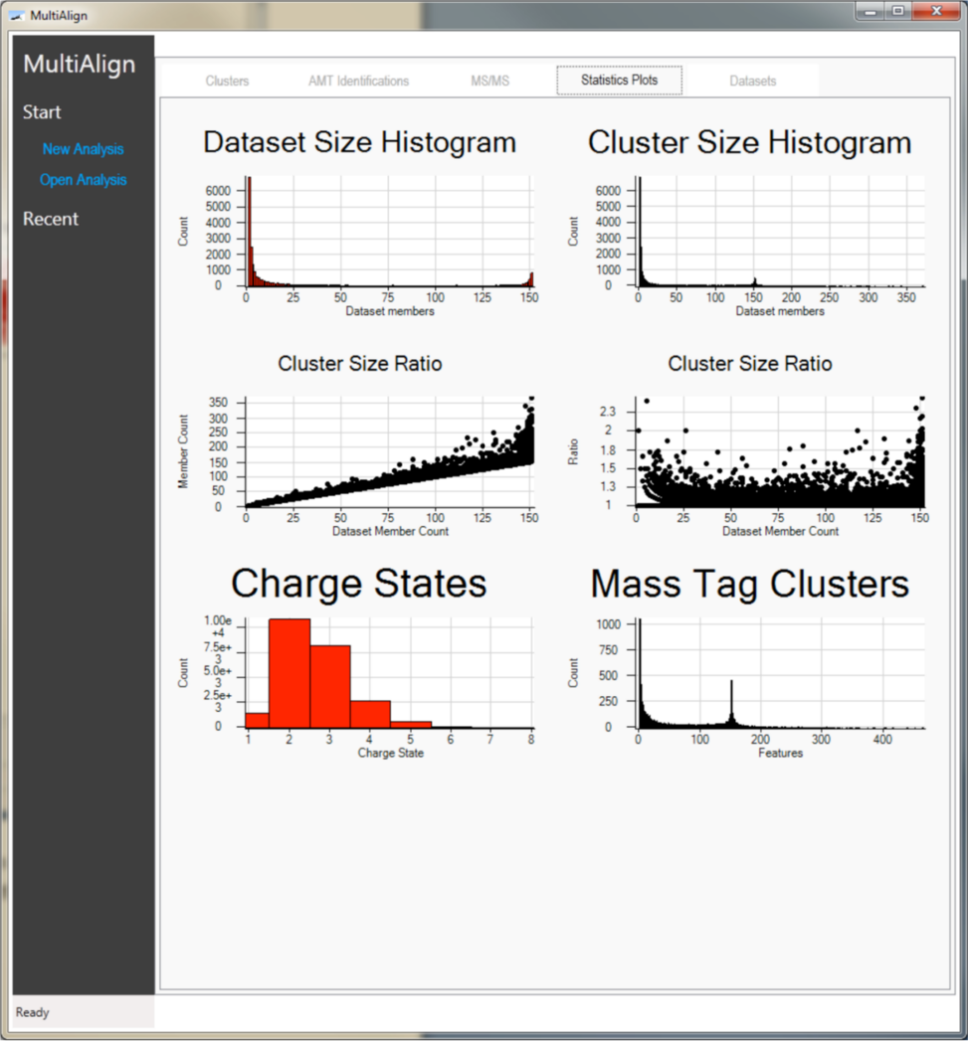
**The Statistics Plots page shows thumbnails of the visualization capabilities in the GUI version of MultiAlign.** This figure shows all plots generated when an AMT analysis is run. Each plot is interactive on this view.

**Figure 5:**
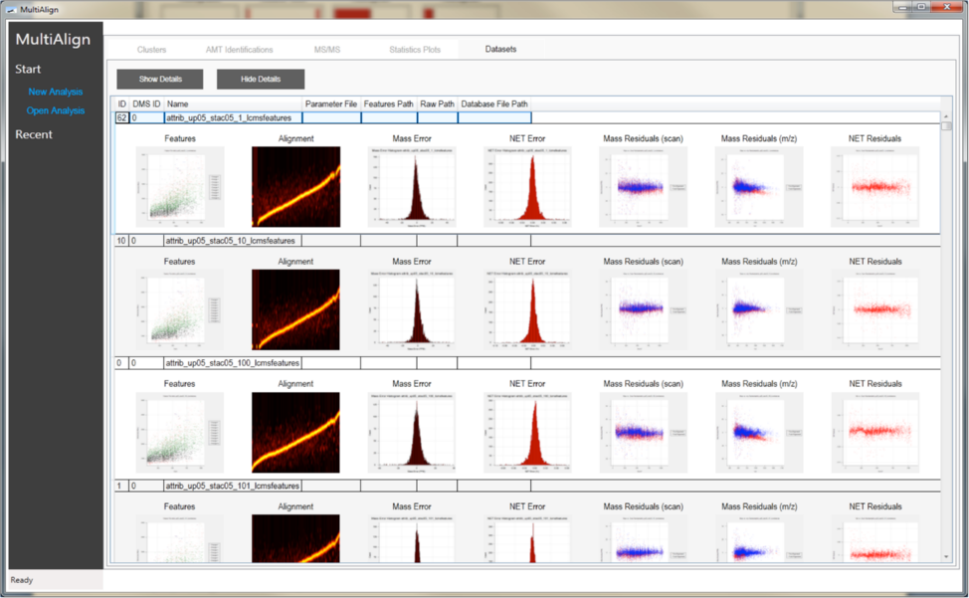
The Dataset Plots page shows all of the thumbnails generated for each dataset to display the alignment plots and feature scatter plots.

**Figure 6:**
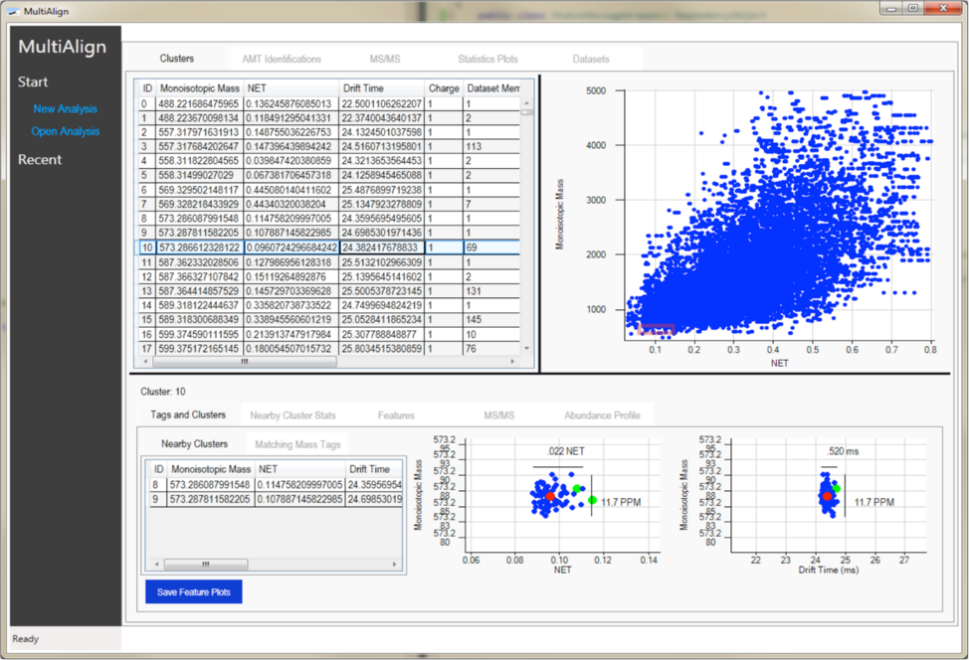
**A scatter plot of all the clusters found in the analysis.** The bottom portion of the screen shows details for the selected given cluster.

MultiAlign has several visualization capabilities, including a fully interactive graphical user interface (GUI). The GUI parameter file editor (Figure 3) can be used to edit parameter files, as well as to export parameters to hyper text markup language (HTML) for easier review. MultiAlign also provides a fully functional GUI application that includes a wizard to guide the researcher through the analysis steps. The wizard is a series of views from which to load data from a data management system [21] (user generated) or local disk, load setup algorithm parameters, and define the output folder. When the analysis is complete, results are displayed in a data view window that facilitates further investigation of features, clusters, and matched peptides when using an AMT tag database. The data view displays all of the results along with interactive plots (for global statistics) that show feature distributions, cluster scores, and error histograms (Figures 4, 5, 6, respectively). The data view also provides an interface to review the parameters used and includes summary information about the number of features found, clusters identified, and peptide sequences identified. Lastly, a report is generated in HTML that contains the plots generated in the data view. This report is packaged with the analysis file and provides users with a way of distributing a data overview to others.

### Parameters

Parameters for all algorithms can be modified through the user interface in the analysis wizard or in the standalone parameter file editor. They also can be adjusted, persisted, and/or loaded in the XML formatted parameter file described in Additional file 1: Table S1. Parameter options include feature loading filters for isotopic fit scores and abundances. Feature finding parameters include mass tolerance (ppm) and scan tolerances.

### File formats

MultiAlign data input formats are in DeconTools “isos” CSV format, ICR2LS “pek” CSV format, and in an LCMS feature tab delimited text file of clustered Decon2LS results. Two forms of data output files are available:

1. **SQLite Database:** This file is an open source-based formatted database that stores pertinent analysis data, and is the main file export format for analysis. The database allows the full traceback to occur from a cluster of features across datasets to an associated MS/MS spectrum. The database schema is published in the Data Tutorial hosted on the project website (http://omics.pnl.gov/software/MultiAlign.php) under the tutorials section.

2. **Cross Tab:** This format is a flat comma separated variable (CSV) file whose rows are clusters and whose columns are attributes about the cluster and features that comprise it.

## Results and discussion

MultiAlign was applied to two environmental proteomics datasets and to a metabolomics dataset to demonstrate its different operational modes. The first application involved a re-analysis of published data [13] acquired from planktonic biomass for which a pseudo metagenome sequence and an AMT tag database were available. In the second application, MultiAlign was used to analyze datasets acquired previously for a microbial community biomass that did not have a metagenomics sequence. These two applications demonstrated the dataset-to-database and dataset-to-dataset alignment capabilities of the software. We also analyzed datasets from a metabolomics study of mitochondrial fatty acid, which demonstrated the applicability of the software to other LC-MS-based omics datasets.

### Proteomics dataset-to-database alignment

MultiAlign was initially applied to re-analyze 24 LC-MS datasets of digested proteins extracted from a groundwater monitoring well sample [13,14]. The sample was collected during acetate addition to a Uranium(VI) contaminated aquifer during a biostimulation field experiment conducted at the U. S. Department of Energy’s Integrated Field Research Challenge site (Rifle, CO). The proteomics data were generated using an LTQ-Orbitrap mass spectrometer (ThermoFisher Scientific Corp., San Jose, CA) operated in HMS-MS^n^ mode (low resolution MS/MS spectra generated from a portion of high-mass accurate parent ions) and coupled to an on-line reverse phase separation of peptides using HPLC. Detailed operating conditions of both the mass spectrometer and HPLC system are described elsewhere [9]. Deisotoped LC-MS spectra [5] were analyzed using MultiAlign and VIPER [8] for comparison. The LC-MS features were peak matched to an AMT tag database that previously had been generated using LC-MS/MS spectra from the 24 datasets and a concatenated set of bacterial genome sequences for iron reducing bacteria [9].

**Figure 7:**
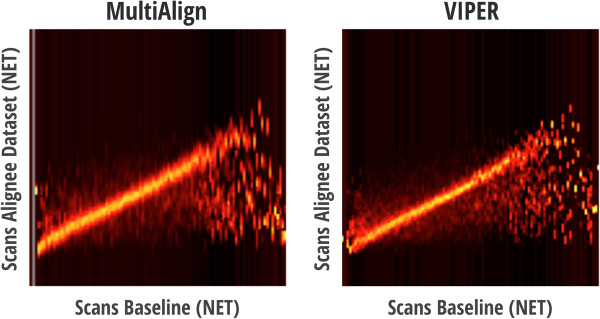
Alignment heat maps for MultiAlign and VIPER of a dataset to an AMT tag database.

Results from the LC-MS feature finding algorithm in MultiAlign ranged from 22,000 to 26,000 LC-MS features per dataset, which is comparable to feature counts per dataset obtained using VIPER. Features identified from both software tools were aligned to mass and NET information in the AMT tag database that represented 17,052 fully tryptic peptides [13,14]. Retention time alignment was performed in a pair-wise fashion, whereby each dataset was aligned to the database. Alignment heat maps for both software tools demonstrate comparable analyses (Figure 7). Noticeable differences between these heat maps may reflect differences in the feature sets as a result of the different LC-MS feature finding algorithms and/or the plotting tools used to create the heat maps. Furthermore, the noticeable deviant line observed in many of the heat maps for both MultiAlign and VIPER indicates potential differences between the database and each dataset [13,14]. This deviance in alignment most likely originates from matches that are similar in mass, but differ in peptide sequences; a common occurrence when analyzing microbial community samples because the database derived from the metagenomics sequence is not comprehensive.

Following this initial comparison, MultiAlign was used to cluster aligned LC-MS features across datasets, after which the cluster centroids were matched to the AMT tag database. In this analysis 37,386 non-singleton clusters (i.e., spanning at least two datasets or more) were observed; however, only 5346 clusters matched unambiguously (STAC uniqueness probability > 0.5) to a single database peptide, which left approximately 86% of the clusters not conclusively identified. A total of 1812 clusters (consisting of both identified and unidentified clusters) had features present across all 12 LC-MS instrument runs. To increase confidence as to which clusters were real, we removed clusters that had an ambiguity score <1, which left 539 clusters with LC-MS features present across all datasets. Of these clusters, only 137 had unambiguous matches to database peptides, leaving 402 unidentified clusters for further investigation.

The small number of unambiguous clusters with features present in all datasets suggests a large difference in both temporal and spatial conditions, which supports previous observations that suggested large differences in community structure in both temporal and spatial conditions [13]. Unlike VIPER, MultiAlign has the added functionality of being able to look across all datasets and then peak match to a database to confidently identify features. Furthermore, when there is no database available, MultiAlign can discover features present across some or all conditions to guide further experimental development.

### Proteomics dataset to dataset alignment and traceback to MS/MS spectra

MultiAlign was also applied to proteomics datasets acquired from an anaerobic microbial community capable of degrading microcrystalline cellulose (Avicel). The community was grown in a bioreactor seeded with biomass obtained from cow rumen. Proteins extracted from five samples harvested 1, 4, 9, 17, and 20 days after bioreactor start-up were digested and analyzed using LC-MS with an LTQ-Orbitrap Velos mass spectrometer (Thermo-Fisher Scientific Corp.) and custom four column HPLC (built in-house). Liquid chromatographic separations were performed using 3-μm Jupiter C18 stationary phase (Phenomenex, Torrence, CA) packed into a 70-cm length of 360 μm o.d. × 75 μm i.d. fused silica capillary tubing (Polymicro Technologies Inc., Phoenix, AZ). The column was equilibrated with 99% mobile phase A (0.1% formic acid in water) and 1% mobile phase B (0.1% formic acid in acetonitrile) prior to sample injection onto a 5 μL sample loop. Automated switching of valve positions transferred the sample to a solid phase extraction column (SPE) for 7 minutes at flow rate of 1.2 μL/min. Four minutes after switching the SPE in line with the analytical column, a programmed gradient ramped the concentration of mobile phase B to 85% over 95 minutes. Column flow was maintained at 0.3 μL/min. Peptides were ionized using an electrospray ionization interface (manufactured in-house). Spectra were collected in MS/MS mode using collision induced dissociation (CID) fragmentation. Each of the five samples was analyzed in triplicate on the instrument and in randomized order on the same chromatographic column. As no metagenomics data were available for the bioreactor community (and thus no AMT tag database could be created), global proteome profiles were compared using MutltiAlign’s dataset-to-dataset alignment and LC-MS feature clustering capabilities, and the parameters in Additional file 1: Table S1.

Results from MultiAlign’s LC-MS feature finding algorithm ranged from 16,500 to 26,000 LC-MS features for the datasets of which 4700 to 9700 matched to MS/MS spectra based on precursor *m/z* and local scans (i.e., MutliAlign’s traceback capability). The unmatched features represent approximately 75% of LC-MS features unidentifiable through traditional database search techniques. The LC-MS feature clustering algorithm found 67,172 clusters with members present in at least two datasets, of which 7098 were unambiguous observations and 110 were present across all time points and in all technical replicates.

**Figure 8:**
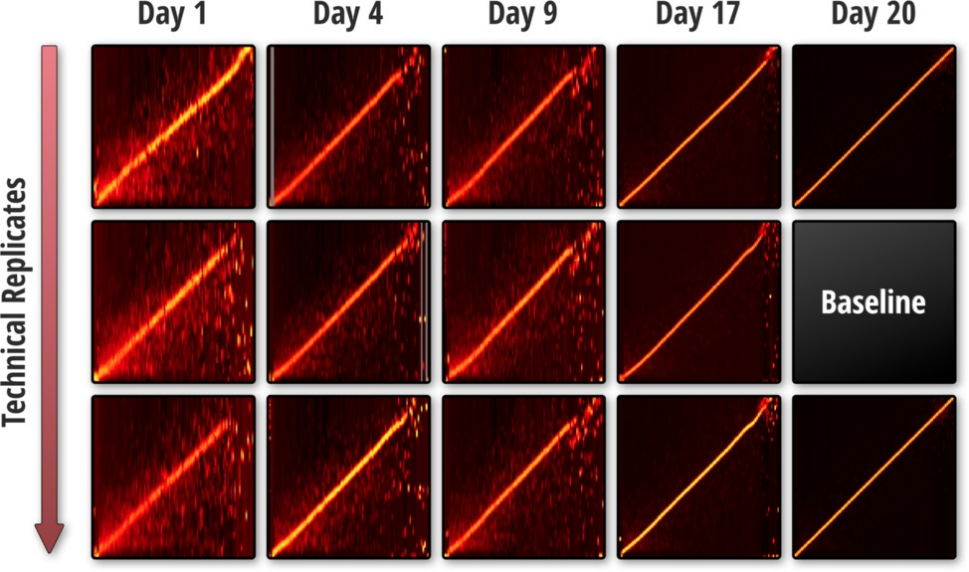
**Bioreactor sample heat-maps aligned to the baseline sample.** Columns represent the day the bioreactor was sampled, rows represent each technical replicate.

While excellent reproducibility was observed for LC retention and mass alignment of technical replicates, alignments at the sample scale (1, 4, 9, 17, 20 days) exhibited increases in retention time and mass variation over the time course (Figure 8). A systematic worsening in alignment was observed regardless of the sample and baseline dataset selected for pair-wise alignment, even though alignment reproducibility was good for technical replicates. Because the run order of samples and technical replicates was randomized, bias introduced from the instrument was ruled out as a cause for this trend.

**Figure 9:**
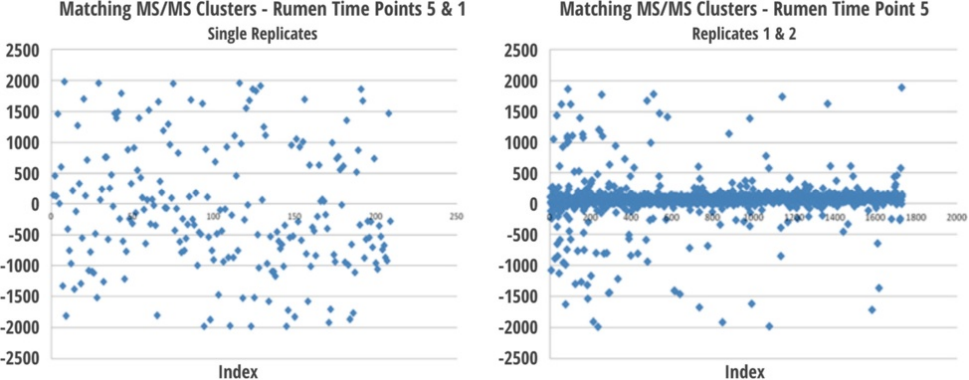
**The delta scans between MS/MS clusters are shown above.** Left, two datasets from day 1 and day 20 from the anaerobic microbial community extracted from cow rumen fluid were clustered using the MS/MS spectral clustering algorithm. Each point represents a spectral cluster. Right, spectral clustering was performed using two datasets from the same sample day. The spectral clustering method shows a significant difference in community structure between sample days one and five (i.e., days 1 and 20).

We performed spectral clustering to better understand the systematic decrease in alignment across time, using MS/MS spectra collected on days 1 and 20 and between two, day-20 technical replicates. Clusters of MS/MS spectra were analyzed and the LC-scan difference between two spectra for each cluster was plotted with respect to the cluster index (Figure 9). Note the differences in both the number and spread of clusters between the two cluster analyses. For the technical replicates, delta scan residuals are close to zero, while delta scan residuals between days 20 and 1 are incongruous, which suggests a lack of similarity between MS/MS spectra generated for these two samples.

Both the cluster analysis plots and the alignment heat maps point to substantial proteome differences in the bioreactor community during its acclimation following inoculation with cow rumen. It is highly likely that both the community structure and proteome were behaving in a dynamic manner during acclimation, which suggests that genomics sequence information derived from any one sample may not represent the proteome of another sample. As a result, a database centric approach would be lacking in that a single metagenome may not capture the entire community structure. Instead, by decoupling this alignment and peptide identification from a database centric model, MultiAlign allows data analysis to be performed on global proteome profiles, without initial genomics sequence information.

### Metabolomics dataset to dataset alignment and traceback to MS/MS spectra

We also demonstrated application of MultiAlign for processing 20 LC-MS-based metabolomics datasets obtained in a previously published study of wild type and mitochondrial fatty acid oxidation enzyme (dodecenoyl coenzyme A delta isomerase) knockdown mutants of human hepatocarcinoma cells [22]. Each dataset contained 4300 to 4900 LC-MS features, and MS/MS traceback results showed 7300 to 8500 MS/MS spectra were matched to these features, which indicated that each LC-MS feature had multiple fragmentation spectra. This observation is in contrast to proteomics datasets, where a single LC-MS feature typically is selected for zero to 1 fragmentation events on average because of sample complexity. In total, 92,730 unique LC-MS features were detected following chromatographic alignment using MultiAlign. The distribution of charge states was predominantly 1+, which is expected in LC-MS metabolomics datasets.

**Figure 10:**
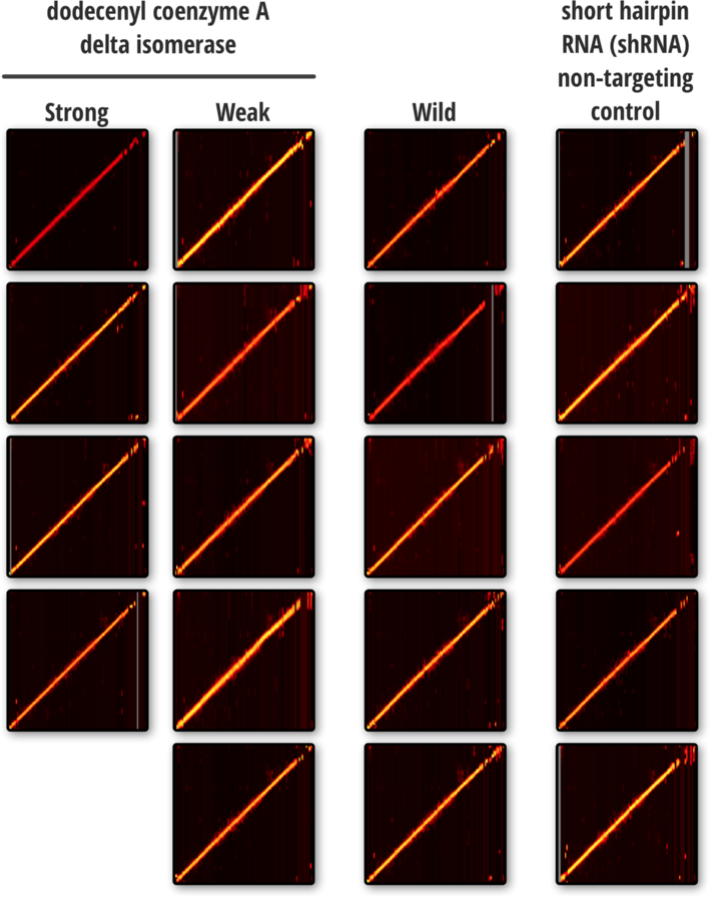
**Alignment heat maps showing similarity between lipidomics datasets from four Huh7 cell experiment groups.** From left to right, the first two groups represent strong and weak knockdowns of *dodecenoyl coenzyme A delta isomerase* expression. The third corresponds to Huh7 wild type cells, and the last is a short hairpin RNA (shRNA) non-targeting control.

**Figure 11:**
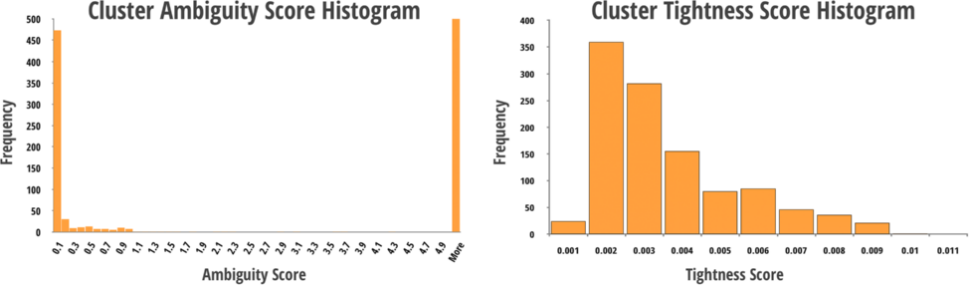
**The histograms above shows the distribution of cluster scores for ambiguity and tolerance using the metabolomics data.** The ambiguity score is the distance between any two clusters. Low scores signify high ambiguity and potential feature overlap between clusters, where high scores would indicate low ambiguity.

Features from individual LC-MS datasets aligned to an arbitrarily chosen baseline dataset resulted in very linear heat maps (Figure 10), with tight mass and NET error distributions (< 5 ppm mass and < 3% NET). After alignment, these features were clustered across datasets, which produced an interesting trend in the cluster size distributions. Of the 92,730 features, we observed 6543 clusters of which 1575 were singletons, i.e. observed in only a single LC-MS dataset. However, we also observed 1089 clusters that spanned all 20 datasets for both cell conditions (i.e., wild type and knockdown mutants). Further investigation of these clusters showed that roughly half (498) had a tightness score < 0.005 and an ambiguity score > 5. The significance of these cutoffs is demonstrated by the two significantly large score distributions in Figure 11.

While we did not quantitatively analyze the 20 metabolomics datasets, we did demonstrate that intensity profiles can be obtained as a result of clustering across conditions. Furthermore, the traceback capability allows researchers to extract MS/MS spectra targets with the intent of comparing them to other metabolomics databases of known standards (e.g., LipidMaps [23]), thereby providing a unique capability for processing LC-MS-based metabolomics data.

## Conclusions

MultiAlign is a new software tool that aligns LC-MS(/MS) feature maps for label-free proteomics and metabolomics comparative analyses. Although capable of matching LC-MS features from a single dataset to a reference database, its value lies in its ability to align multiple datasets to find differentially abundant chemical species of interest not readily identified by traditional approaches, which allows for focused identification. This alignment capability supports targeted workflows for identifying systematic changes in complex samples, such as microbial communities, to assist downstream investigations of underlying biological processes.

When reference databases are available, clustered species derived from multiple datasets can be matched to entries in a database and assigned confidence values, after which selected species can be followed up using a targeted experimental approach, e.g. using selected reaction monitoring to validate identifications or more MS/MS spectra collection with other fragmentation methods such as CID, HCD, or ETD. Both identified and unidentified clusters can also be profiled to reveal abundance changes, which is useful for supporting or establishing hypotheses for cause/effect, spatial, or temporal studies. Furthermore, MultiAlign is useful for studies where little to no genomic context is known and reference databases cannot be constructed, as exemplified by our application of the software to proteomic samples of an anaerobic microbial community. In this analysis we demonstrated how two dissimilar LC-MS feature maps could be analyzed using MultiAlign’s MS/MS traceback and spectral clustering capability. This functionality allows researchers to focus further informatics investigation, e.g. *de novo* peptide identification approaches, on MS/MS spectra that are associated to features found in multiple experiments, as opposed to transient spectra.

## Availability and requirements

**Project name:** MultiAlign

**Project home page:**http://omics.pnl.gov/software/MultiAlign.php

**Operating system(s):** Microsoft Windows XP and newer (32-bit and 64-bit)

**Programming language:** C# for presentation layer (visualization) and C++ for computation layer.

**Other requirements:** Microsoft .NET framework 4.0, Microsoft .NET framework 2.0

**License:** Apache 2.0

**Any restrictions to use by non-academics:** None.

## Abbreviations

MS: Mass spectrometry; LC-MS: Liquid chromatography-mass spectrometry; AMT: Accurate mass and time (related to accurate mass and time tag proteomics)

## Competing interests

The authors declare that they have no competing interests.

## Authors’ contributions

BLL developed visualization capabilities and improved performance of back-end algorithmic components, as well as improved scalability and application to targeted workflows. BLL also wrote several of the data access components, and provided critical bug fixes. KC developed data access layer components for input and output and upgrades to the user interface, and provided support and testing for various platforms. KC also played a role in critical bug fixes. AS and VP provided algorithmic oversight through code reviews and played a role in testing and analysis of data. NJ developed initial versions of the software, including the underlying alignment, feature finding, clustering, and peak matching algorithms. GK and JS wrote database access tools. MEM provided feature development and access to AMT-DB creation utilities and provides testing and data analysis. SJC provided guidance for targeted workflow development, feature development, and application of MultiAlign for environmental proteomics analyses. TM provided metabolomics datasets and direction for LC-MS analysis of metabolites. GAA directed development of the tool towards various application areas for high-throughput LC-MS analysis. RDS provided crucial scientific guidance and leadership to the development group.

## Supplementary Material

Additional file 1: Table S1 Table of parameters used in the MultiAlign analysis. Click here for file
