# Supplementary material for: MultiAlign: a multiple LC-MS analysis tool for targeted omics analysis
Source: BMC Bioinformatics. 2013 Feb 12;14:49. doi: 10.1186/1471-2105-14-49 (PMC3599190; doi:10.1186/1471-2105-14-49)
Supplement: Additional file 1: Table S1 — Table of parameters used in the MultiAlign analysis. [file 1471-2105-14-49-S1.pdf]

MS-MSn Feature Linking			
Category	Parameter Name	Value	Description
Tolerances	MZTolerance	0.15	The m/z tolerance window to match an MS feature to an MS/MS spectra based on its precursor mass

LC-MS Feature Finding			
Category	Parameter Name	Value	Description
Filtering	IsIsotopicPeakFitFilterInverted	False	Flag indicating if the isotopic fit score is inverted. False would mean that a score = 0 represents a good fit, score = 1 representing a poor fit. True would indicate the inverse.
Filtering	IsotopicFitFilter	0.15	Fit score filter.
Filtering	IsotopicIntensityFilter	0	Average Mass Constraint (ppm) to look between MS Features for similarity.
Filtering	MaxDistance	0.1	Maximum distance two MS Features could be considered. Distance is considered based on mass, NET, fit score, intensity.
Filtering	MinUMCLength	3	The minimum number of scans a LC-MS feature can be observed across.
Filtering	UseIsotopicPeakFitFilter	True	Determines if MS Features should be filtered based on their isotopic fit values.
Filtering	UseIsotopicPeakIntensityFilter	False	Determines if MS features should be screened by their intensity values.
Intensity/Abundance	UMCAbundanceReportingType	Sum	Determines how to report the abundance of a LC-MS Feature.
Splitting	SplitFeatures	True	Determines if features should be split after feature finding. True is recommended.
Weights	AveMassWeight	0.01	Average mass weight.
Weights	ConstraintAveMass	6	Average Mass Constraint (ppm) to look between MS Features for similarity.
Weights	ConstraintMonoMass	6	Monoisotopic Mass Constraint (ppm) to look between MS Features for similarity.
Weights	FitWeight	0.1	Deisotoping Fit Score Weight.
Weights	LogAbundanceWeight	0.1	Weight for the abundance (log base 2 transformed).
Weights	MonoMassWeight	0.01	Monoisotopic mass weight.
Weights	NetWeight	0.1	Normalized Elution Time (NET) weight.
Weights	ScanWeight	0.01	Scan weight.

LC-MS Filtering			
Category	Parameter Name	Value	Description
Charge States	MaximumChargeState	6	Maximum Charge State a feature can have.
Charge States	MinimumChargeState	1	Minimum Charge State a feature can have.
Elution Time	MinimumScanLength	3	Minimum Scan Length a feature can span.
Intensity/Abundance	MinimumAbundance	0	Minimum abundance allowed.
Mass	MaximumMonoisotopicMass	100000	Maximum Monoisotopic Mass a feature can be.
Mass	MinimumMonoisotopicMass	0	Minimum Monoisotopic mass a feature can be.
Scores	MinimumIsotopicFit	0.15	Minimum isotopic fit score

Mass Tag Database			
Category	Parameter Name	Value	Description
Experiments	ExperimentFilter		Experiment Filter
Experiments	ExperimentExclusionFilter		Experiment Exlusion Filter

Ion Mobility	OnlyLoadTagsWithDriftTime	False	If True, only mass tags with drift time > 0 will be loaded.
Scores	MinimumXCorr	0	Minimum X-Correlation
Scores	MinimumObservationCountFilter	0	Minimum MS-MS Observations
Scores	MinimumPMTScore	3	Minimum PMT Score
Scores	MinimumDiscriminant	0	Minimum Discriminant
Scores	PeptideProphetVal	0.99	Prophet Value
Tags	ConfirmedTags	False	Determines if only those tags that were confirmed are loaded.
Tags	NETValType	0	How NET will be used. 0 for global average NET (recommended), 1 for predicted NET

LC-MS Feature Alignment			
Category	Parameter Name	Value	Description
Alignment Function	MassCalibrationLSQNumKnots	12	Determines how many least square knots should be used.
Binning	HistogramDriftTimeBinSize	0.03	Histogram size of alignment (ms)
Binning	HistogramNETBinSize	0.001	Bin size for NET error histograms.
Binning	HistogramMassBinSize	0.2	Histogram size of alignment in parts per million (PPM)
General Calibration	AlignmentType	NET_MASS_WARP	Determines if NET only, or Mass and NET alignment should be performed.
General Calibration	RecalibrationType	HYBRID_CALIB	Type of recalibration to perform, NET only, or NET and Mass
General Calibration	SplitAlignmentMZ	False	Determines whether the m/z boundaries should be used for analysis. False if recommended.
Mass Calibration	MassCalibrationLSQZScore	2.5	Determines the z-score cutoff for mass calibration
Mass Calibration	MassCalibrationMaxJump	20	
Mass Calibration	MassCalibrationMaxZScore	3	
Mass Calibration	MassCalibrationNumMassDeltaBins	50	Number of divisions to make in the mass dimension.
Mass Calibration	MassCalibrationNumXSlices	12	Number of divisions to make in NET.
Mass Calibration	MassCalibrationUseLSQ	False	Determines whether least squares is used for the fit.
Mass Calibration	MassCalibrationWindow	20	Mass Calibration window to use in parts per million (PPM)
Matching	MaxPromiscuity	3	Total number of candidate matches that can be allowed for a single LC-MS Features before being discarded.
Matching	UsePromiscuousPoints	False	Determines whether features can match to multiple features or whether they are considered ambiguous.
Tolerances	MassTolerance	20	Mass tolerance in parts per million (PPM)
Tolerances	MaxTimeJump	10	Largest scan range allowed for a feature match.
Tolerances	NETTolerance	0.05	NET tolerance for allowable feature matches.
Tolerances	NumTimeSections	100	Number of divisions to make in NET.
Weights	ContractionFactor	2	Determines how far away a baseline scan can be compared to alignee scans.

Drift Time Alignment - IMS

Category	Parameter Name	Value	Description
Alignment	ShouldPerformOffset	False	Should perform offset correction based on error distributions.
Alignment	UseAllObservationsForOffset	False	
Ion Mobility	ShouldAlignDriftTimes	False	Determines if drift time alignment should be computed at all. True yes = IMS, False no = LC-MS.
Tolerances	DriftTimeTolerance	5	Drift time tolerance (ms).
Tolerances	MassTolerance	15	Monoisotopic mass tolerance in parts per million (PPM)
Tolerances	MaxChargeState	4	Maximum charge state to consider
Tolerances	MinChargeState	1	Minimum charge state to consider.
Tolerances	NETTolerance	0.03	Normalized elution time (NET) tolerance.

LC-MS Feature Clustering			
Category	Parameter Name	Value	Description
AMT	AlignClusters	False	This is only valid if you have a mass tag database. Setting to True will align each cluster to the database.
Centroid	ClusterRepresentativeType	Mean	Determines how the centroid should be calculated.
Ion Mobility	IgnoreCharge	True	Set Ignore Charge to True if you are not using Ion Mobility (IMS). Set to False if you are using IMS.
Tolerances	DriftTimeTolerance	1000	For Ion Mobility Data. Tolerance in drift time (ms) set to a large value if not using LC-MS
Tolerances	MassTolerance	6	Mass Tolerance in parts per million (PPM).
Tolerances	NETTolerance	0.014	Normalized elution time (NET) tolerance.

Peptide Identification - STAC			
Category	Parameter Name	Value	Description
AMT	UsePriors	True	Determines if prior probilities of the tags being matched to should be used.
AMT	WriteResultsBackToMTS	False	Determines if results from STAC should be written back to the Mass Tag System (PNNL Only).
Binning	HistogramBinWidth	0	Determines the width of the bin
Binning	HistogramMultiplier	0	
FDR Calculation	ShiftAmount	11	Shift amount for the dalton shift FDR calculation.
FDR Calculation	ShouldCalculateHistogramFDR	False	Determines if a FDR histogram should be calculated.
FDR Calculation	ShouldCalculateShiftFDR	False	Determines if STAC should calculate the dalton shift FDR.
FDR Calculation	ShouldCalculateSLiC	False	Determines if STAC should calculate The SLiC Score previously used by VIPER
FDR Calculation	ShouldCalculateSTAC	True	Determines if the STAC scores should be calculated
Ion Mobility	UseDriftTime	False	Determines if Ion Mobility was used.
Tolerances	UseEllipsoid	True	
Tolerances	DriftTimeTolerance	100	Drift time tolerance if using Ion Mobility data.
Tolerances	MassTolerancePPM	25	Monoisotopic Mass tolerance in parts per million (PPM).
Tolerances	NETTolerance	0.035	Normalized elution time (NET) tolerance.
Tolerances	Refined	True	Determines if tolerances were refined using STAC.

Feature Consolidator			
Category	Parameter Name	Value	Description
Abundance	AbundanceType	Sum	Determines how the abundance from features of the same dataset in a cluster will be reported. Either by summing their abundance, or by taking the maximum

Table 1: Table of parameters used in the MultiAlign analysis.
